# A comparative evaluation of frictional resistance and surface roughness of silver coated and uncoated stainless-steel bracket wire assembly- An *in-vitro* study

**DOI:** 10.4317/jced.60357

**Published:** 2023-05-01

**Authors:** Pooja Shah, Padmaja Sharma, Harshit Naik, Kalpesh Patel, Chetan Panchal

**Affiliations:** 1 Department of Orthodontics and Dentofacial Orthopaedics, Manubhai Patel Dental College and Hospital and ORI, Vadodara, Gujarat, India; 2 Maharaja Sayajirao University, Vadodara, Gujarat, India

## Abstract

**Background:**

Silver ions act as potent antimicrobial agents. Silver coating of brackets and the archwires can help reduce the formation of white spot lesions and caries which is commonly seen with fixed orthodontic treatment. However, this may affect the friction and surface roughness of the bracket-wire assembly which in turn affects the biological tooth movement.

**Material and Methods:**

A total of 60 samples were included in the study which was divided into four groups. Group-1: • 15 silver coated 0.022 x 0.028” slot MBT prescription maxillary central incisor brackets • 15 silver coated 0.019 x 0.025” stainless-steel wires; Group-2: • 15 uncoated 0.022 x 0.028” slot MBT prescription maxillary central incisor brackets • 15 silver coated 0.019 x 0.025” stainless-steel wires; Group-3: • 15 silver coated 0.022 x 0.028” slot MBT prescription maxillary central incisor brackets • 15 uncoated 0.019 x 0.025” stainless-steel wires; Group-4: • 15 uncoated 0.022x0.028” slot MBT prescription maxillary central incisor brackets • 15 uncoated 0.019 x 0.025” stainless-steel wires. All brackets and wires used were of American Orthodontics, St. Paul, USA. Surface modification of wires and brackets was carried out using the Vacuum Coating Unit model by Thermal Vacuum Evaporation method with silver nanoparticles (10 nm size). The frictional resistance of all brackets and wires was checked using Universal Testing Machine.

**Results:**

On comparison of maximum load, it was found that friction was highest in group 3, followed by group 1, group 4 and group 2. The mean difference between all groups was found to be statistically significant with a *P* value < 0.05. The Scanning Electron Microscope studies showed that the surface roughness of silver-coated wires and brackets before the friction test was less compared to uncoated wire-bracket assembly. The surface roughness of the bracket and wire after the friction test was as follows: •Bracket roughness: Group 4> Group 1> Group 2> Group 3 •Wire roughness: Group 4> Group 1> Group 2> Group 3.

**Conclusions:**

This study concluded that friction was least when only the wire was coated with silver and the bracket was uncoated and it was the most when the bracket was coated and the wire was uncoated. The surface roughness after the friction test was the least when the wire was uncoated.

** Key words:**Silver nanoparticles, Frictional Resistance, Surface Roughness.

## Introduction

The main purpose of orthodontic treatment by fixed appliances is to align the teeth thereby correcting their function and enhancing their aesthetics. The fixed appliances mainly include bands, brackets, arch wires and auxiliaries. The design and surface characteristics of these appliances influence plaque retention. Moreover, these appliances make it difficult for patients to achieve proper brushing and oral hygiene maintenance, which increases the caries incidence (1,2). The mouth harbors the most diverse bacterial communities in the human body and a unique variation in oral microbiome structure is observed according to the different surface properties of the oral structures (3). The complicated undercuts on the orthodontic appliances lead to plaque accumulation which changes the oral microbiome thereby increasing the risk of enamel demineralization, white spot formation, dental caries, gingival inflammation, and periodontal disease. The issue of bacterial infection can be solved by adjusting the antimicrobial properties of the surface of the metal prior to appliance application (4). Various metals have been used for centuries as bactericidal and bacteriostatic agents. Amongst them, silver ions or salts are well known for their antimicrobial effects since ancient times (5). Active silver ion is non-toxic to mammalian cells but is active against numerous primitive bacterial forms, fungi, or viruses. Thus, it seems an exotic choice to be used as an antimicrobial agent (5). Considering the effects of silver ions on plaque accumulation in fixed appliance therapy and the effects of frictional forces and surface roughness on the orthodontic tooth movement, the assessment, and comparison of surface topography and frictional resistance of the silver ion coated and uncoated bracket wire system is important. The comparison of frictional resistance and surface roughness of silver-coated and uncoated wires and silver-coated and uncoated brackets has been done separately in various studies (6-8). However, to the best of our knowledge, no study has been carried out on coating both the brackets and wire simultaneously with the silver ion. Hence, this study focuses on the comparison of frictional resistance and surface roughness of coated and uncoated bracket wire assembly.

## Material and Methods

-Study design and participants

This in-vitro study was carried out in the institute and was approved by the Institutional Research and Ethical Committee. The selection criteria included (1) 0.022 × 0.028” slots of McLaughin Bennett Trevisi (MBT) prescription central incisor brackets (Maxillary central incisor brackets, American Orthodontics, United States) and (2) 0.019 × 0.025” SS wires (American Orthodontics, United States). The defective wires and brackets were excluded.

-Study sample size

The sample size was estimated using the sample size calculator. A minimum of 30 units (15 silver-coated brackets and 15 silver-coated wires and 15 brackets and wires without coating) were included in the present study to estimate the mean difference of frictional force 0.33 N between two groups with SD 0.265 N at 99 % confidence and 80 % power.

-Procedure of surface modification of stainless-steel wires and brackets with silver nanoparticles

**Figure 1:**
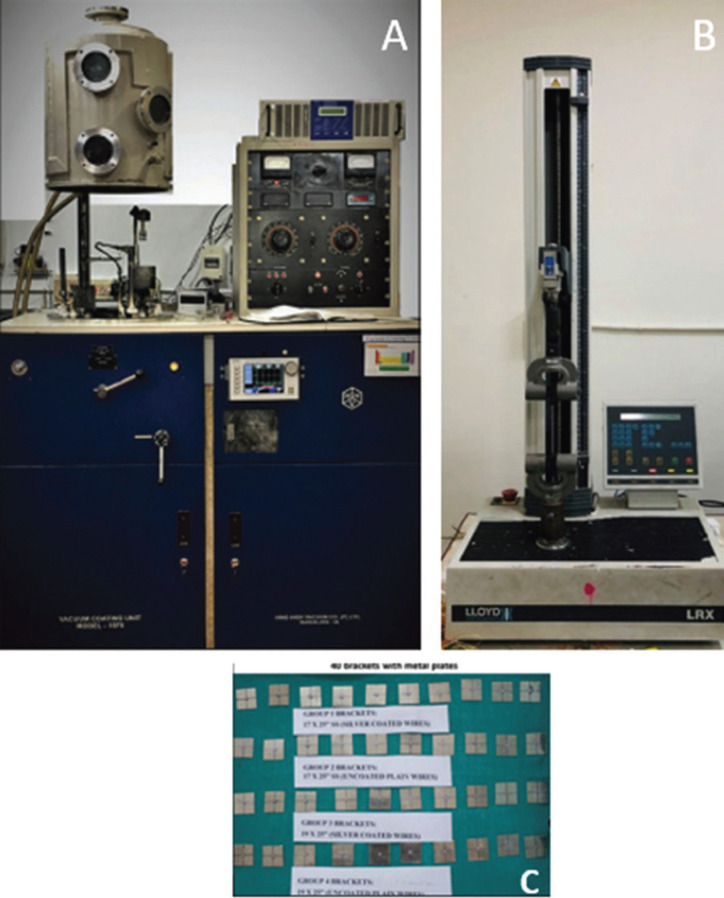
(A) Vacuum-Coating Unit Model, HINDHIVAC Vacuum-Coating Unit Model no. 15 F6 (Hind High Vacuum Co., Bangalore), helps in producing fine uniform, pure film coating of various metals; (B & C) Metal plates with testing apparatus- (B) central incisor brackets mounted on metal plates and (C) universal testing machine is used for checking frictional resistance.

The vacuum coating instrument HINDHIVAC (Hind High Vacuum Co., Bangalore) Vacuum Coating Unit Model no-15 F6 (Fig. 1) was used for coating brackets and wires. It produces homogeneous, thin, pure, uniform film coatings of various metals. This instrument has facilities for ion cleaning (bombardment), thermal evaporation, etc., with accessories for rotation, substrate heating, film thickness monitoring, etc. In this study, thermal evaporation method was used for Surface modification of Stainless Steel wires and brackets. A thin coating of 99.9% pure silver was done on orthodontic brackets and wires. These appliances were coated with a uniform thickness of 10nm which was measured in-situ using a quartz crystal thickness monitor. The gas used for sputtering was argon (Ar) gas (7 sccm.) and its flow was controlled by the mass flow controller (MFC, AALBORG, Germany). Sputtering is a technique where the target material to be used as the coating is bombarded with ionized gas molecules causing atoms to be “sputtered” off into the plasma. These vaporized atoms are then deposited as a thin film on the substrate to be coated. In this study, 40 W DC sputtering power and  10 milli-torr pressure were used.

In this study, the thickness of the silver nanoparticle film was kept at 10 nm to avoid any significant alteration in its dimension. Thirty Stainless Steel sheets were cut in sizes of one inch wide and one inch in length. The horizontal and vertical reference lines were drawn on the plate. Central incisor brackets were attached at the intersecting point of these reference lines. The experimental procedure was carried out under dry conditions and at room temperature using a Universal Testing Machine (UTM) as shown in Fig. 1. The SS plate with the bracket was attached to the friction testing device. The SS plate with the bracket was fixed to the lower arm of UTM. The straight part of the posterior segment of the wire was cut up to 20 mm. This straight segment of each wire was then ligated to the bracket with the ligature wire. One end of the wire was fixed in the upper arm of UTM and the other end of the wire was placed in the slot and secured with ligature wire. The load cell registered the force levels required to move the wire along the bracket; this level was then transferred to a computer hard disk.

The wires were moved on the bracket at a crosshead speed of 5mm/min. The unit for calculating load values of frictional resistance was Newton (N). After each test, the testing machine was stopped, the bracket archwire combination was removed and a new wire bracket assembly was placed and tied with a new ligature wire and the frictional resistance was tested. This experiment was done for all 4 groups ( Table 1) i.e., silver-coated wire and bracket (Group 1), silver-coated wire and uncoated bracket (Group 2), silver-coated bracket and uncoated stainless-steel wire (Group 3), and uncoated wire and bracket (Group 4).

**Table 1:**
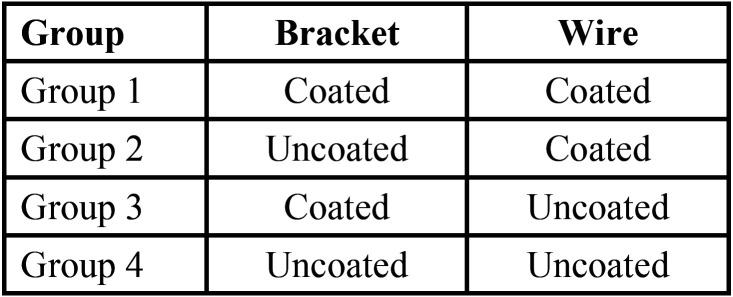
Grouped samples.

## Results

The descriptive statistics of the variables under study showed that the mean load at the limit was 3.047 N and the average maximum load was 6.955 N. Deflection at the limit was almost the same for all the groups with the lowest standard deviation value of 0.040 respectively ( Table 2).

**Table 2:**
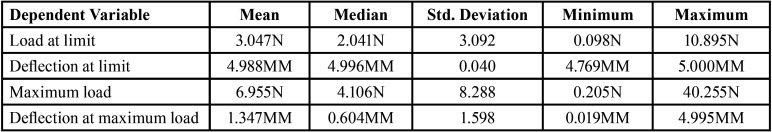
Descriptive Statistics.

The surface roughness of 2 bracket wire assembly, randomly picked up from each group was checked using Scanning Electron Microscope at 100x (coated and uncoated brackets and wires before friction test and coated and uncoated brackets after friction test) and 150x (coated and uncoated wires after friction test) magnification at 20kV.

A comparison of maximum load using one way ANOVA test showed that load at the limit, maximum load, and deflection at maximum load had statistically significant differences between the four groups under study. On considering load at the limit, it was seen that Group 1 had higher friction, followed by Group 4. For maximum load, again Group 3 had the highest friction followed by Group 1. However, considering deflection at maximum load it was seen that, the friction was higher for Group 2 ( Table 3). On comparison of maximum load, it was found that the friction was higher in Group 3, followed by Group 1, Group 4, and then Group 2 respectively.

**Table 3:**
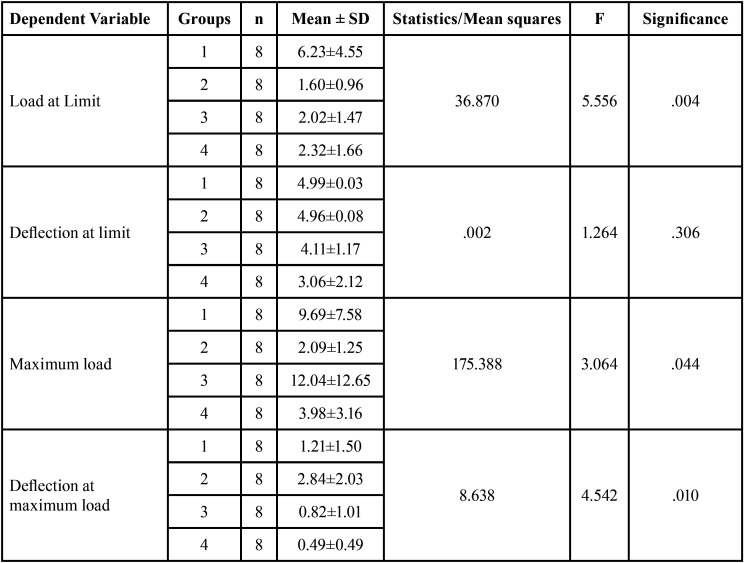
One-way ANOVA with post hoc Tukey test for comparison of the four group values.

Post hoc Tukey test ( Table 4) showed that for load at the limit, the difference between Group 1 and Group 2, Group 1 and Group 3, and Group 1 and Group 4 were statistically significant with a mean difference of 4.6 N, 4.2 N and 3.9 N with *p-value* < 0.05. However, for maximum load, findings showed that the mean difference between all the groups was found to be statistically significant with *p* value < 0.05 respectively.

**Table 4:**
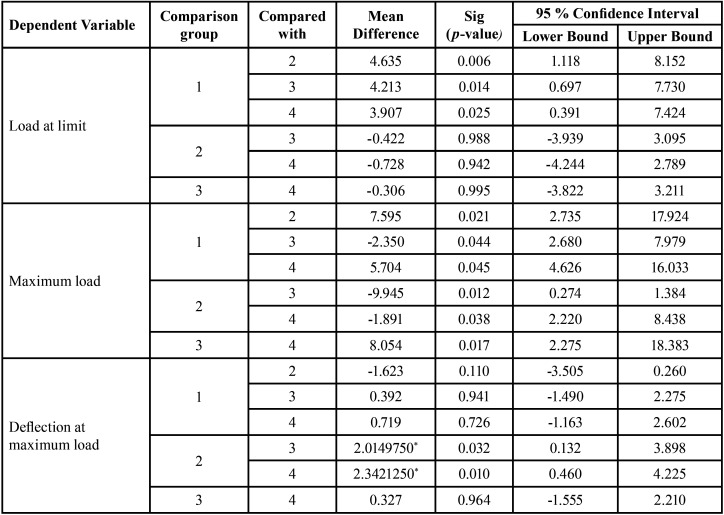
Post Hoc Tukey test for intergroup comparison.

**Figure 2:**
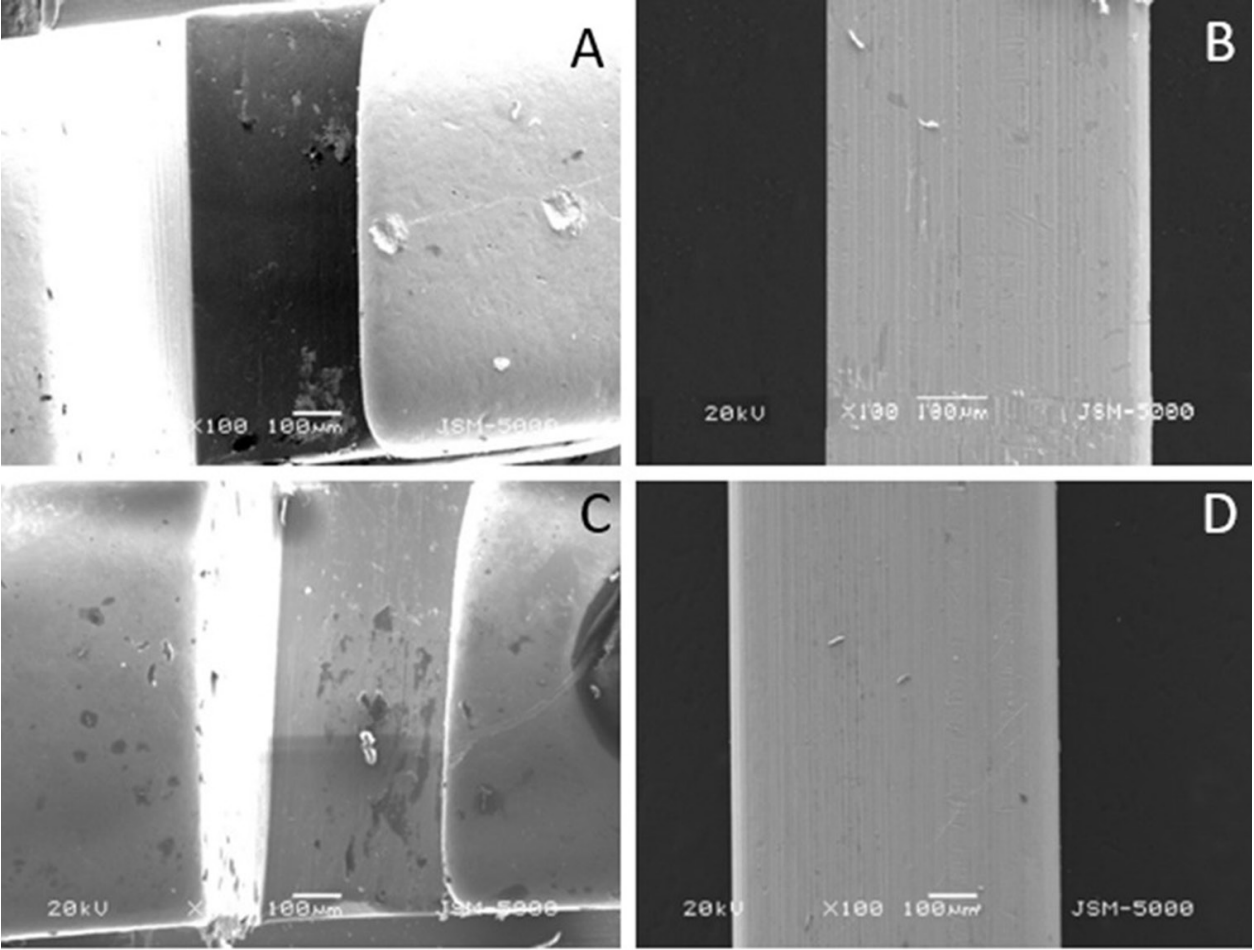
(A). Coated Bracket before friction; (B). Coated Wire before friction; (C). Uncoated Bracket before friction; D. Uncoated Wire before friction.

**Figure 3:**
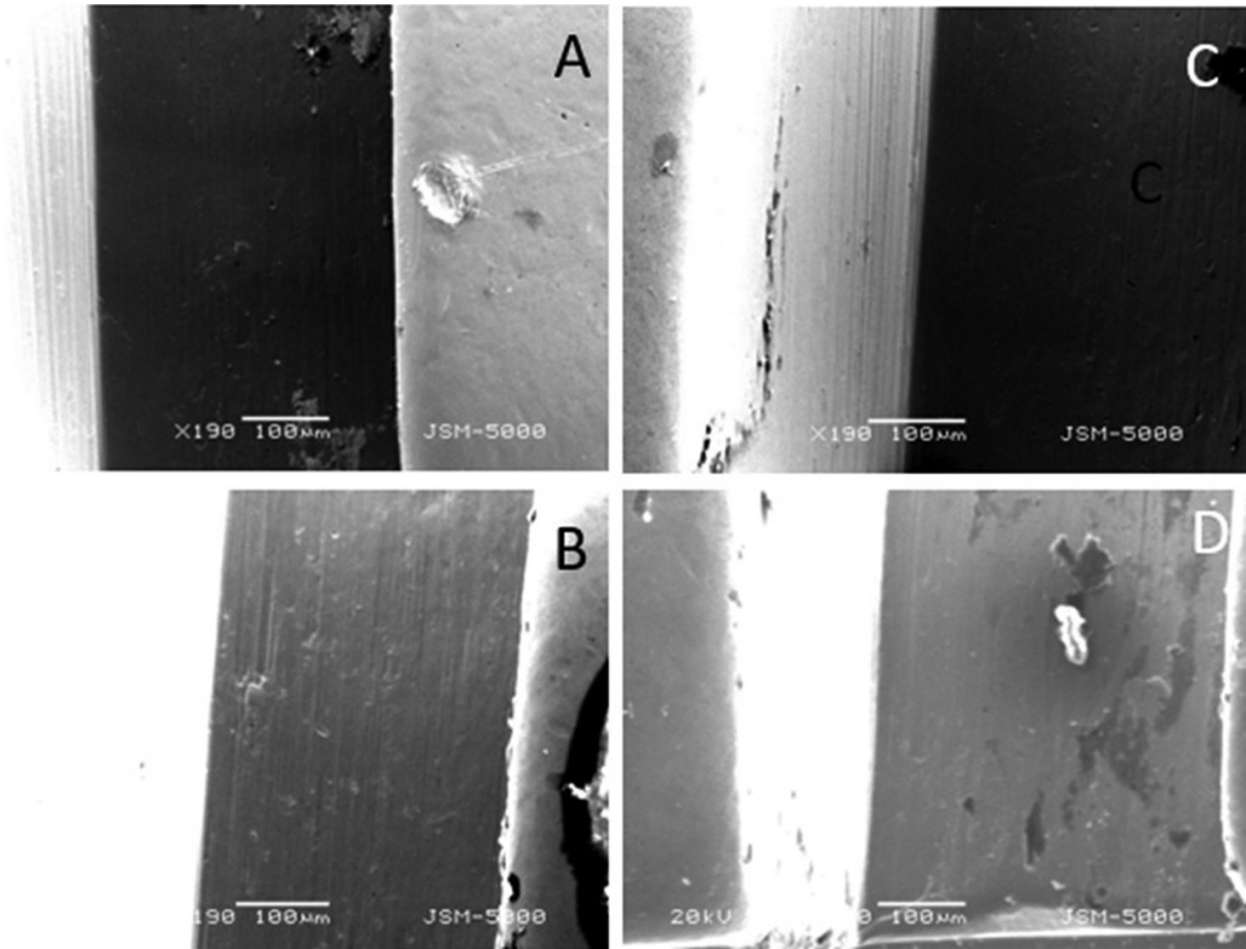
(A) Group 1: Coated Bracket; (B) Group 2: Uncoated Bracket; (C) Group 3: Coated Bracket; (D) Group 4: Uncoated Bracket.

**Figure 4:**
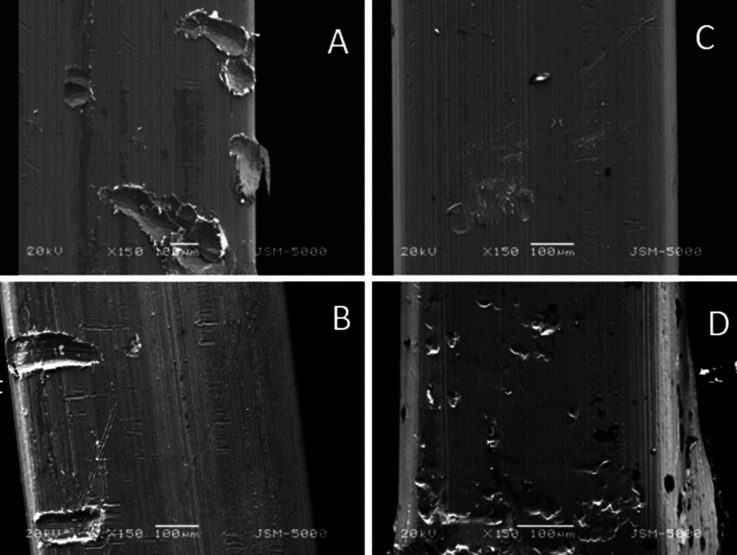
(A) Group 1: Coated Wire; (B) Group 2: Coated Wire; (C) Group 3: Uncoated Wire; (D) Group 4: Uncoated Wire.

The SEM studies showed that the surface roughness of silver-coated wires and brackets before the friction test (Fig. 2) was less compared to the uncoated wire-bracket assembly. Whereas the surface roughness of the bracket and wire after the friction test was as follows (Figs. 3,4).

Bracket roughness: Group 4> Group 1> Group 2> Group 3 

Wire roughness: Group 4> Group 1> Group 2> Group 3

## Discussion

The fixed orthodontic appliances accelerate the plaque accumulation rate which in turn causes enamel decalcification and gingival inflammation. Various studies (9,10) have found a relationship between the bacteria in the oral cavity and fixed orthodontic treatment. It was noted that there is an increase in the incidence of dental caries and *S. mutans* bacterial counts with fixed orthodontic appliances. Not only cariogenic bacteria, the colonization of periodontal pathogenic bacteria such as *P. nigrescens*, *P. intermedia*, *P. gingivalis*, and *F. nucleatum* was also found with the placement of orthodontic appliances (3).

Adhesion of bacteria to the surface of fixed orthodontic appliances is an extremely complicated process that is affected by many factors like environmental factors, bacterial properties and the material surface characteristics such as bonding structure, surface charge, chemical composition, hydrophobicity and topography-roughness. Wang *et al*. (2). pointed out that carbon films prevent bacterial adhesion and several authors (11-14) have also reported that carbon films show antibacterial properties.

Surface modification with a suitable element such as gold, platinum, silver, zinc, copper, or a combination of them has been proven to greatly increase antibacterial properties. Amongst them, silver is known to be the most effective antibacterial agent since ancient times in medicine and has been used in many different forms in biomedical engineering with good effects (nanoparticles, nanocomposite, colloids, foams, polymers, fibers, etc.) to hinder the biofilm formation and hence the incidence of infections (15). Silver is also effective against a large range of fungi and viruses.

Metals and their alloys present with various physical and mechanical properties. Amongst them, corrosion and elemental release are some of the major disadvantages of metals when used as biomaterials. Saliva, as it contains bacteria, viruses, yeast, fungi, and their products may cause corrosion of orthodontic appliances. The alloys used in orthodontic appliances rely on the formation of a passive oxide film to resist corrosion, but this layer can be disrupted by chemical and mechanical attacks. Moreover, orthodontic treatment with fixed appliances provides a unique environment for the colonization of microorganisms since these devices contain structural irregularities that make it difficult for patients to maintain adequate oral hygiene (6).

Additional challenges like friction are also encountered when using metals as orthodontic biomaterials. Frictional control is a major obstacle since a percentage of the applied force is dissipated to overcome friction, while the remaining percentage is transmitted to the supporting structures to induce tooth movement.

The amount of archwire deformation is dependent on the applied force and elasticity of the wire. Some of the applied force is used to overcome the friction between the bracket and the archwire. It is therefore conferred that materials with low coefficients of friction should be used for straight wire mechanics as they can reduce the strain on anchorage and hence reduce the force levels on the tooth. Numerous factors have an impact on friction. Besides the alloy composition of the archwire, the elasticity, the wire size, and the surface structure including surface treatments also play an important role. In situ studies have shown the influence of both friction and biocompatibility on the surface characteristics of archwires. Plaque accumulation is affected by the surface roughness which in turn affects the surface properties of the wire (16).

In order to overcome such weaknesses, various kinds of research have been conducted by coating the surface of the archwire with different metals such as silver, gold, zinc, molybdenum, etc., to render these materials more suitable for orthodontic applications. Literature shows that surface treatment of the archwires reduces friction by up to 46 % (17).

Arash *et al*. (4) in one of their studies, electroplated the brackets with silver films of 8–10 micrometer thickness and did not find any significant improvements in the frictional resistance. They hypothesized that the reason why friction was not reduced was due to the increased thickness of the deposited silver layer. However, with the advent of nanoscience, pure silver can now be converted into nanometer-sized particles which helps us use the various benefits of pure silver (10) and enables the coating of the ultra-thin layer of silver on the fixed orthodontic appliances. In our study, we have coated the bracket-wire assembly with nanoparticles of silver of 10 nm thickness.

A study by Juan Francisco *et al*. (6) was conducted to compare the antibacterial properties of silver, gold, and zinc nanoparticles against the *S. mutans*. Findings showed that the nanoparticles of silver, as compared with those of gold and zinc oxide, required a lower concentration to inhibit the development of the *S. mutans* strains. These results suggest that silver nanoparticles may be the most effective ions in controlling *S. mutans* and therefore caries. Silver nanoparticles (Ag NPs) are also effective against Gram-positive and Gram-negative bacteria, some antibiotic-resistant strains, and also, against fungi and viruses. Hence, in our study, we coated the wire-bracket assembly with silver nanoparticles.

Gamze *et al*. (18) performed an *in vivo* study on nano silver-coated orthodontic brackets placed on the mandibular incisors of Wistar Albino rats to evaluate the antibacterial properties and ion release from the coated brackets. The study showed that the nano silver-coated orthodontic brackets favoured the inhibition of the *S. mutans* and reduction of caries on smooth surfaces which suggested that these brackets act as an antibacterial agent without patient compliance and could be helpful for the prevention of white spot lesions during fixed orthodontic treatment.

Ameli *et al*. (8) investigated the effects of orthodontic brackets coated by silver hydroxyapatite (S-HAP), copper oxide (CuO), and titanium oxide (TiO2) nanoparticles on wire-bracket friction. In this in-vitro study, the friction between wires (Stainless Steel and Nickel-Titanium) and brackets were compared. The study concluded that coating brackets with TiO2 and CuO nanoparticles can reduce friction. Moreover, Niti round wires show the least friction as compared to rectangular or round Stainless Steel wires with all types of brackets.

Mathew *et al*. (19) reviewed the effect of nanoparticle coatings on frictional resistance (FR) of orthodontic archwires in a systematic review and Meta-analysis. A total of ten *in vitro* studies were included in the qualitative analysis and five studies were included in the quantitative analysis for this review; eight of the included articles identified a significant decrease in FR of the coated wires when compared to uncoated wires. There was a significant reduction in FR of Nanoparticle (NP) coated SS wires when compared with uncoated wires. Hence both qualitative and quantitative assessments of the available literature suggested a significant reduction in FR of orthodontic archwires subjected to NP coating.

The antibacterial activity of silver nanoparticles is influenced by their size. There is an increase in biocompatibility and stability with a decrease in their size. The smaller Ag-NPs have a higher surface area-to-volume ratio, which allows them to readily penetrate biological surfaces. Therefore, the smaller Ag-NPs are more toxic than the larger particles.

The mechanism of action of silver involves the continuous release of nanoparticles of silver and the adherence to the cytoplasmic membrane. The adhered ions enhance the permeability of the cytoplasmic membrane and result in the disruption of the bacterial envelope. After the uptake of free silver ions into cells, the respiratory enzymes are often deactivated, generating reactive oxygen species but interrupting ATP production. The interaction of silver ions with the sulphur and phosphorus of DNA can cause problems in DNA replication, and cell reproduction and may even lead to the termination of the microorganism. Moreover, silver ions can inhibit the synthesis of proteins by denaturing ribosomes within the cytoplasm (20).

Various methods can be used to coat silver nanoparticles on the wire-bracket system such as Physical vapor deposition (PVD) (e.g., evaporation and sputtering), electroplating, chemical vapor deposition, atomic layer deposition, spin coating, spray pyrolysis, etc. In our study, the bracket-wire assembly was coated by the sputtering method of Physical Vapour Deposition (PVD) in the Vacuum Coating Unit wherein argon (Ar) gas was used as a sputtering gas. Pure silver (99.9 %) was used to obtain a thin coating on the orthodontic wire and bracket. The advantages of the PVD method are that it is relatively safe and can be used on any inorganic material including orthodontic brackets and wires. PVD coatings are sometimes harder and more corrosion-resistant than coatings applied by electroplating processes (21). Most coatings have high temperatures and good impact strength, excellent abrasion resistance. Moreover, the vacuum environment also provides the ability to reduce gaseous contamination in the deposition system to a low level. The disadvantages of coating by PVD are higher cost and the process requiring complex machines that need skilled operators.

As the efficiency of fixed appliance therapy depends on the fraction of force delivered with relevance to the force applied, high frictional forces resulting from the interaction between the bracket and therefore the guiding arch-wire affect the treatment outcomes and duration in a negative way. Methods and properties of arch-wire ligation have a very important role in generating friction. Most investigations (22-24) have concluded that elastomeric modules significantly increase the resistance to sliding compared to the stainless-steel ligatures, especially when the latter are tied loosely. Hence in our study, we’ve ligated the wire to the bracket with 0.008 mm thick stainless steel ligature wire.

The results of this study suggested that the friction was seen higher when the bracket was coated with silver nanoparticles and the wire was uncoated (Group 1), whereas the friction was lowest in the group with an uncoated bracket and coated wire (Group 2). The friction of the bracket-wire assembly in descending order was as follows: Group 3> Group 1> Group 4> Group 2.

The surface roughness of the bracket and wire after the friction test was as follows: Bracket roughness: Group 4> Group 1> Group 2> Group 3.

Wire roughness: Group 4> Group 1> Group 2> Group 3.

In our study, when the bracket and wire were coated with silver nanoparticles, it was found that the surface roughness and the frictional resistance both were high after the friction test was done. This could be because of the wearing off of the surface coating when the wires slid against the bracket.

When the wire was coated and the bracket was uncoated, the surface roughness was less and the frictional resistance of the same was reduced. This shows the shearing forces when the coated wire was slid against the uncoated bracket, which causes less wearing off, of the coating.

When both the brackets and wires were uncoated, the surface roughness was the highest compared to the frictional resistance. This could be due to the increased shearing forces between the uncoated surfaces. This shows that the coating reduces the frictional resistance.

This infers that the surface roughness and frictional resistance are the lowest when only the wire is coated, and the bracket is uncoated whereas the frictional resistance was highest when only the bracket was coated with silver ions. However, since the friction is not only affected by the degree of surface roughness but also by the geometry of the roughness, orientation of roughness features, passive surface film, and the relative hardness of the two contacting surfaces (22), the variation between the surface roughness and the frictional resistance among the different groups needs further investigations and studies to be justified.

Orally administered silver in ionic and nanoparticulate forms has been described to be deposited in a wide range of organs. Owing to advances in nanotechnology, nanoparticles potentially decrease the required dosages while increasing safety through reduced side effects. In a study by Van der Zande *et al*. (25), less deposition was observed following silver nanoparticle administration than following ionic silver administration.

Our study was *in-vitro* and was focused on reducing the chances of white spot lesions and bacterial adhesion thereby reducing the incidence of caries which is most commonly seen in fixed orthodontic therapy. Hence, we have coated the bracket and wire with silver nanoparticles which is a good antibacterial agent. However, with every alteration made to the bracket-wire assembly, there will be changes in their properties like friction and surface roughness, which will in turn affect the orthodontic tooth movement. Moreover, in the mouth, the situation is much more complex and bracket archwire interaction varies continuously, which can be stated as the limitation of our study. Also, further studies are required regarding the toxicity of silver when used *in-vivo* and more *in-vivo* evidence is needed regarding its antimicrobial effects.

## Conclusions

The results of the study suggested that:

• The friction was highest in group 3 (Coated bracket and uncoated wire), followed by group 1 (Coated bracket and wire), group 4 (Uncoated bracket and wire), and was least in group 2 (Uncoated bracket and coated wire).

• The SEM studies suggested that the surface roughness of silver-coated wires and brackets before the friction test was less compared to the uncoated wire-bracket assembly.

• The surface roughness of the bracket and wire after the friction test was as follows: (i) Bracket roughness: Group 4> Group 1> Group 2> Group 3 (ii) Wire roughness: Group 4> Group 1> Group 2> Group 3.

Hence, we can conclude that the coating of brackets and archwire improves the surface roughness, but frictional resistance appears to be much more reduced when only the wire is coated with silver nanoparticles.
